# CT imaging of dogs with perineal hernia reveals large prostates with morphological and spatial abnormalities

**DOI:** 10.1111/vru.13087

**Published:** 2022-03-29

**Authors:** Tuuli M. Åhlberg, Hanna M Salonen, Outi M Laitinen‐Vapaavuori, Sari H Mölsä

**Affiliations:** ^1^ Department of Equine and Small Animal Medicine, Faculty of Veterinary Medicine University of Helsinki Helsinki Finland

**Keywords:** canine, computed tomography, Osirix®, prostate volume

## Abstract

The etiology of canine perineal hernia (PH) remains unclear, although as a disease of older male dogs, it is likely to be hormonal. The role of the prostate in the formation of PH has been questioned; however, prospective and systematic evaluation of prostates in these dogs is absent in the literature. In this prospective case–control study, CT imaging was used to assess prostatic changes in dogs with PH (n = 46) and compare these findings with those of intact age‐matched male dogs (n = 23). Using the OsiriX® DICOM viewer, we measured prostatic volume and correlated it with the size of the dog by using the length of the sixth lumbar vertebra. In addition, we recorded spatial and morphological changes of the prostate, such as heterogenicity, intra‐ and paraprostatic cysts, and mineralizations, as well as prostatic location and rotation. We found that dogs with PH had larger prostates (*P* < .001) that more often contained cysts (*P* < .001) and had larger cyst diameters (*P* = .013) than age‐matched controls. Prostates of PH dogs also contained paraprostatic cysts (17.4%) and focal mineralizations (32.6%), which were absent in the control group. Abnormal rotation and location of the prostate were common in dogs with PH. In conclusion, these findings support the use of CT as an adjunct diagnostic imaging modality for the evaluation of the prostate in dogs with PH. Further studies are needed to evaluate nonprostatic CT findings in the pelvic cavity of PH dogs.

**Abbreviations**
BPH: benign prostatic hyperplasia
CT: computed tomography
DICOM: Digital Imaging and Communication in Medicine
MPR: multiplanar reformatting
PH: perineal hernia
RV: ratio volume
VTHUH: Veterinary Teaching Hospital of the University of Helsinki

## 1 INTRODUCTION

Perineal hernia (PH) is a disease of older mainly intact male dogs that is notable for its unclear etiology.^1^, ^2^ The role of the prostate in PH etiopathogenesis is debated, with some studies questioning its contribution and others suggesting a possible association with the disease.^3^, ^4^, ^5^, ^6^ Volumetric, morphological, and spatial features of the prostates in PH dogs remain undefined. Establishing these features is needed to assess the role of the prostate in PH etiology. As most published studies on PH are retrospective and focus on surgical management and outcome, diagnostic imaging has not been routinely used in the assessment of PH. Prostatic changes have been reported variably as incidental findings on rectal palpation, radiographs, ultrasonography, or visualization during PH surgery.^4^, ^6^, ^7^ Rectal palpation and radiographic imaging as methods for detecting prostatic disease are common but relatively unreliable.^8^, ^9^ Ultrasound examination, the current gold standard for examining prostatic size and morphology, can be challenging in dogs with PH because of the prostate's location.^7^, ^8^, ^9^

Increasing in popularity, CT imaging has been reported to be helpful for evaluating prostatic morphology and size without superimposition of surrounding tissue.^9^, ^10^ Computed tomography also allows for correlation of prostatic volume with the size of the dog by using structures such as the length of the sixth lumbar vertebra (L6).^10^, ^11^ Prostatic size estimations typically involve single parameters such as height, width, and length; volumetric measurements appear, however, to be more accurate and are becoming increasingly available.^10^, ^11^, ^12^ No previously published studies were found assessing the size, morphology and location of the prostate in dogs with PH using CT imaging.

The primary aims of this study were to determine the prostatic volume of dogs with PH using CT imaging and to compare the results with the control group of age‐matched intact male dogs. A secondary aim was to describe the morphological and spatial changes of prostates in dogs with PH. Our primary hypothesis was that dogs with PH have larger prostates than their age‐matched controls. Our secondary hypothesis was that prostates of dogs with PH are heterogeneous, contain abnormal quantities of cysts and mineralizations, and have abnormal location and rotation.

## 2 MATERIALS AND METHODS

### 2.1 Study design and selection of dogs

This case–control study consisted of two groups of male dogs, a prospective group suffering from PH (PH group) and a retrospective group gathered from the Veterinary Teaching Hospital of the University of Helsinki (VTHUH) database (control group).

The PH group included male dogs with naturally occurring PH that were referred to the VTHUH from March 2017 to December 2020. We excluded dogs with previously surgically treated PH, American Society of Anesthesiologists’ physical status rating of 4 or 5, severe renal or hepatic disease, or other diseases that might increase the risk for anesthesia. We also excluded dogs that had been surgically or chemically castrated. Decisions for inclusion and exclusion were made by an ECVS‐certified veterinary surgeon (S.M.). Ethics approval for this research was granted by the Research Permit Board ELLA: ESAVI/4467/04.10.07/2017. Owners gave written consent for their dogs to take part in the study.

The control group gathered from the VTHUH database (Provet Net, Finnish Net Solutions Oy) included intact male dogs aged over 5 years that had undergone CT imaging from August 2016 to December 2020 for various reasons. Only CT images containing the entire prostate and L6 were included. Exclusion criteria comprised chemical and surgical castration and findings of PH on CT images. No ethics approval was required based on the Finnish national legislation, which states that a need for ethics approval or owner's consent is deemed unnecessary for retrospective studies analyzing patient data and images gathered from the electronic database of VTHUH (https://finlex.fi/en/laki/kaannokset/2013/20130497).

### 2.2 CT imaging

In the PH group, the dogs underwent a physical examination and hematological and biochemical blood value analyses to assess health status. Clinical assessments were performed by a PhD researcher in clinical veterinary medicine (T.Å.). The dogs received intramuscular premedication of 0.3 mg/kg methadone (Insistor vet® 10 mg/ml; Richter Pharma AG, Wels, Austria) and 0.02 mg/kg acepromazine (Plegicil® 10 mg/ml; Bela‐Pharm GmbH & Co KG, Vechta, Germany) with anesthesia induced with 1–4 mg/kg propofol to effect (Propovet Multidose 10 mg/ml; Fresenius Kabi AB, Uppsala, Sweden) and maintained with sevoflurane (end‐tidal concentration 2.3%) in oxygen. In the control group, due to the retrospective nature of the study, anesthesia was not standardized.

All dogs underwent CT imaging in a helical 64‐slice multidetector CT scanner (Lightspeed VCT, GE Healthcare, Madison, WI, USA). In the scans of the PH group, the voltage was 120 kV, collimation pitch 0.516, speed 20.62 mm/rotation, rotation time 0.6 s, detector coverage 40 mm, and matrix 512 × 512. An automodulation was used, and the maximum current varied from 650 to 750 mAs. The slice and interval thicknesses were 0.625 mm. Dogs were scanned in dorsal recumbency from the middle of the fourth lumbar vertebra to the most caudal aspect of the dog. All dogs received 2 ml/kg contrast agent (Omnipaque® 300 mg/ml) injected into an intravenous catheter in the vena cephalica. Scans were started one minute after beginning the injection.

Image analysis was performed by two observers (H.S. and T.Å.) using commercially available DICOM image processing software (Osirix®, version 11.0.4, Pixmeo, Bernex, Switzerland). Both observers were licensed veterinarians; one with 12 years of experience in veterinary diagnostic imaging at VTHUH (H.S.) and another PhD researcher in clinical veterinary medicine (T.Å). The prostatic volume was measured by the first observer (H.S. who was blinded to the clinical signs of the dogs. All morphological and spatial changes were recorded by the second observer (T.Å.).

The prostate was evaluated in a soft‐tissue window (window level 40 HU, window width 400 HU). The length of L6 was measured in the bone window (window level 500 HU, window width 3500 HU). All measurements were performed in series with contrast agent, except for nine dogs in the control group in which only noncontrast series were scanned.

### 2.3 Volumetric measurements

The prostate perimeters were manually traced in original transverse images with the “Closed Polygon” tracing tool in each slice that contained prostatic tissue, after which the volume was measured using the “Compute Volume” tool. The urethra as well as any cysts connected to the prostate were included in the volume. Volumetric measurements were performed once. The length of L6 was measured at the shortest part of the vertebra in sagittal multiplanar reformatting (MPR). To correlate prostatic volume with the size of the dog, we calculated the ratio volume (RV) by the following formula: cubic root of the prostatic volume divided by L6 length.^12^ A 95% confidence interval (CI) for prostatic RV in the control group was used to differentiate between a small, medium, and large prostate. We defined prostates as small if they were below the lower CI and large if they exceeded the upper CI. These were used as reference values in the PH group.

### 2.4 Morphological and spatial assessments

Without histological samples, the description of prostatic changes was based on CT images alone. The parenchymal appearance was described as either homogeneous or heterogeneous. As cysts and abscesses appear similar on CT images, focal rounded or irregular hypoattenuating cyst‐like lesions were called intraprostatic cysts. The prevalence (no/yes) and number of intraprostatic cysts over 5 mm in diameter were recorded, and prostates were placed into classes depending on the number of cysts: class 1 (1–5 cysts), class 2 (6–10 cysts), and class 3 (>10 cysts). The diameter of the largest cyst in each prostate was measured. Large, rounded, hypoattenuating cyst‐like lesions that protruded from the prostate were recorded as paraprostatic cysts (no/yes). The maximum diameter was recorded. Focal hyperattenuating lesions were recorded as mineralizations (no/yes), and the quantity of these lesions was recorded.

**FIGURE 1:**
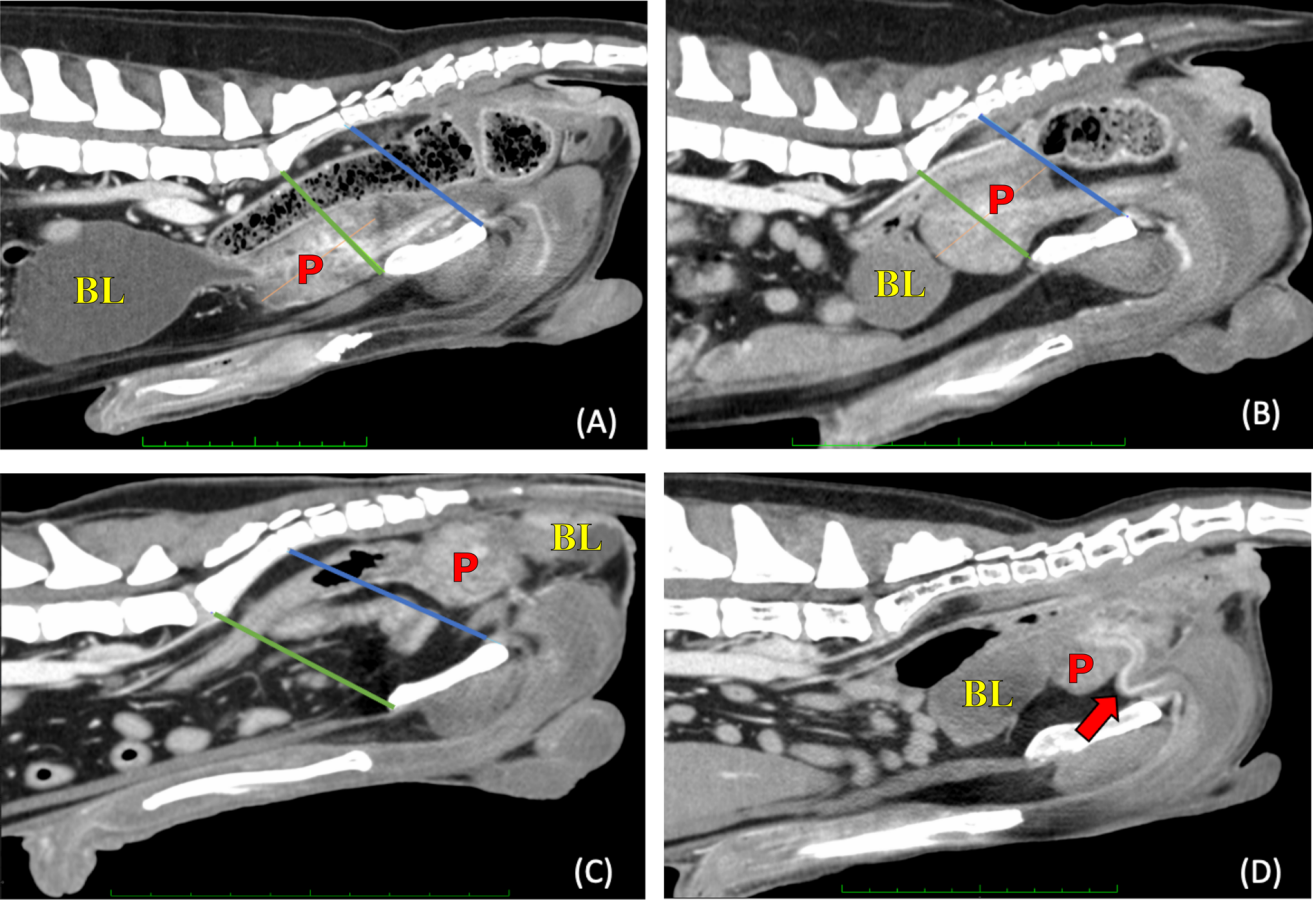
Postcontrast computed tomographic images of the caudal abdomen in sagittal views, demonstrating the cranial (black line) and caudal (white line) borders of the pelvic cavity for measuring the location of the prostate gland (P) and bladder (BL). The prostate is located (A) in the abdomen, (B) in the pelvic cavity, and (C) in the hernia. D, Flexure of the urethra (arrow). Soft tissue reconstruction (window level: 40 HU, window width: 400 HU) and a slice thickness of 0.625 mm were used [Colour figure can be viewed at wileyonlinelibrary.com]

Location of the prostate was defined in midline sagittal MPR. Borders of the pelvic cavity were defined by drawing a line from the most cranioventral aspect of the sacrum to the cranial pubic brim (cranial border) and another from the most caudoventral aspect of the sacrum to the ischial arch (caudal border). The length of the prostate was measured in the same plane. Location was defined by the percentage of the length that crossed either of these borders, visualized in Figure 1A–C. The position of the prostate was defined as abdominal if over 80% of the prostate's length was in front of the cranial border or in the hernia if over 80% was located behind the caudal border. Prostates that did not meet these criteria were described as pelvic.

**FIGURE 2:**
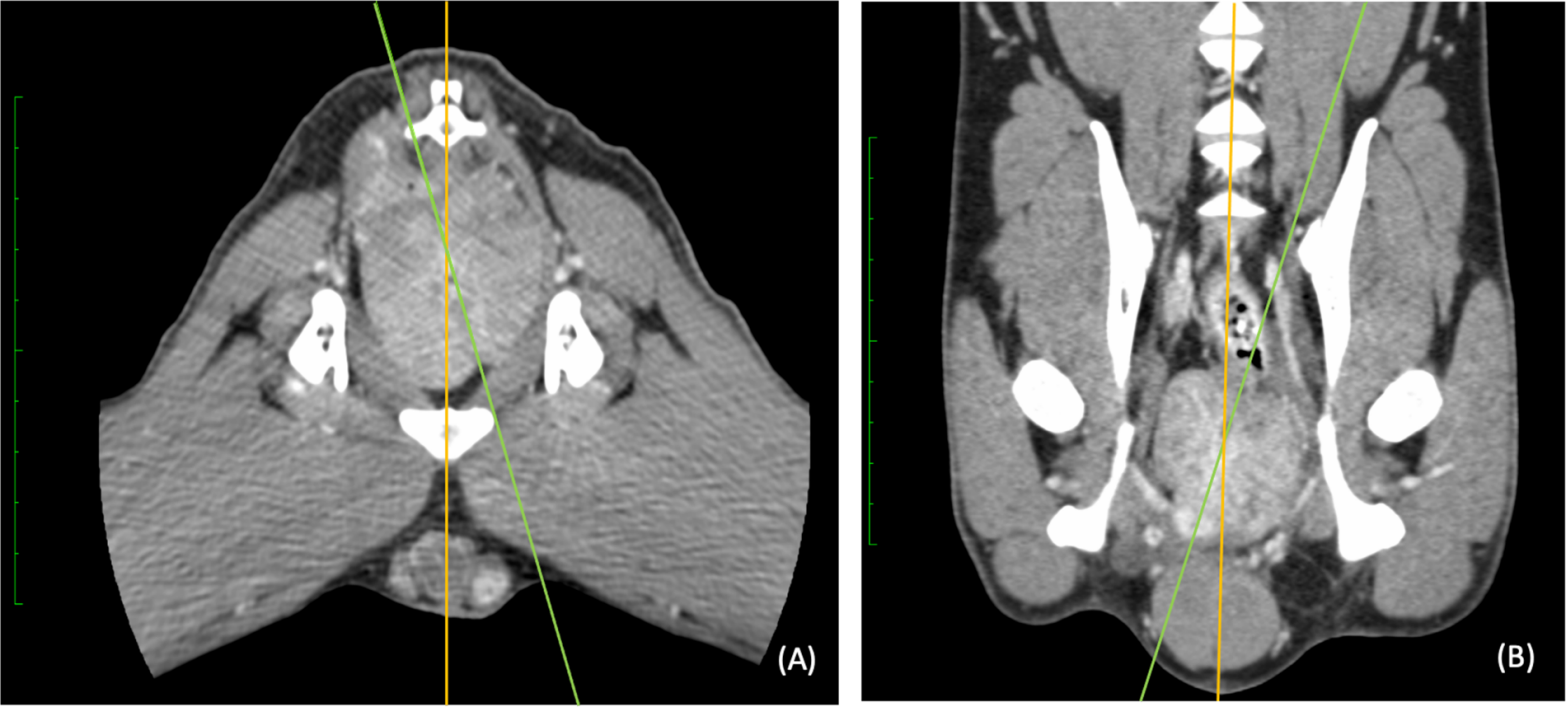
Postcontrast computed tomographic images of the caudal abdomen demonstrating rotation of the prostatic axis (dotted line) measured from (A) the vertical axis (white line) on the transverse image and (B) the longitudinal axis (white line) on the dorsal image. Soft tissue reconstruction (window level: 40 HU, window width: 400 HU) and a slice thickness of 0.625 mm were used [Colour figure can be viewed at wileyonlinelibrary.com]

Rotation of the prostate was described as deviation of the prostatic axis from the midline of the dog, shown in Figure 2A and B. The prostatic axis was defined in dorsal and transverse MPR by drawing a line through the midline (the prostatic groove) of the prostate. The midline of the dog was defined as the longitudinal axis in the dorsal MPR and the vertical axis in the transverse MPR. We measured the size of the angle between the prostatic axis and the longitudinal axis (rotation in the dorsal plane) as well as the angle between the prostatic axis and the vertical axis (rotation in the transverse plane).

The location of the bladder, unlike the prostate, cannot be defined by the location of its body, as its size varies depending on its grade of filling. In our study, bladder location was described as abdominal, pelvic, or located in the hernia, depending on the location of the bladder neck.^13^ Possible S‐shaped cranio‐caudal deviation of the urethra occurring caudal to the ischial arch was recorded (no/yes) (Figure 1).

### 2.5 Statistical analysis

Statistical analysis was performed by a PhD researcher (T.Å.) in collaboration with a statistician. Prostatic volume was analyzed using SAS® System for Windows, version 9.4 (SAS Institute Inc., Cary, NC, USA), and morphological changes were analyzed with IBM® SPSS® Statistics, version 27.0.1 (IBM Corp. ©, Armonk, NY, USA). Descriptive statistics are presented as the mean ± SD (range) values for the continuous variables. Additionally, to determine reference values for prostatic RV, 95% CI was calculated. Frequency values and percentage distributions were used for categorical values.

The differences in prostatic RV between the PH group and the control group were analyzed with an analysis of covariance (ANCOVA) model, where the group was used as a fixed factor and age as a covariate. Model residuals were investigated visually using QQ plots and formally with the Shapiro–Wilk test to detect deviations from model assumptions. The differences in the prevalence of heterogeneity and intraprostatic cysts between the PH and control groups were analyzed with Pearson's chi‐square test, while the mean size of the largest intraprostatic cyst as well as the degree of prostatic rotation between groups were compared with the Mann–Whitney test. *P* values < .05 were considered statistically significant.

## 3 RESULTS

### 3.1 Selected dogs

In the PH group, 46 dogs fulfilled the inclusion criteria. The most common breeds were mixed breed (n = 8), Cotton de Tulear (n = 4), Dachshund (n = 3), Finnish Lapphund (n = 2), Norwegian Elkhound (n = 2), Belgian Shepherd (n = 2), Australian Kelpie (n = 2), and Border Collie (n = 2). The mean ± SD age was 8.1 years ± 1.7 (range 5.1‐11.5) and weight 16.9 kg ± 9.5 (range 4.3‐38.0). Altogether, 23 dogs met the inclusion criteria in the control group. The most common breeds were Shetland sheepdog (n = 7) and Yorkshire terrier (n = 2). The mean ± SD age was 8.0 years ± 2.4 (range 5.1–13.2) and weight 20.3 kg ± 19.5 (range 1.6–62.5).

In the control group, the different protocols used depended on the indication for CT imaging (presented in Supporting Information 1). The voltage was 100–140 kV, collimation pitch 0.516–1.375, speed 20.62–55.00 mm/rotation, rotation time 0.4–0.8 s, detector coverage 40 mm, and matrix 512 × 512. The maximum current varied from 80 to 669 mAs. The slice thickness was 0.625 mm in all dogs except dog #1 (1.25 mm) and dog #8 (2.5 mm). Dogs 1‐14 received 2 ml/kg intravenous contrast agent, and the timing of imaging after injection varied (portal or interstitial phase) depending on the indication.

Indications for CT examination in the control group were suspected neoplasia (n = 8), orthopedic evaluation (n = 7), trauma (n = 2), suspected portosystemic shunt (n = 2), suspected PH (n = 1), back pain (n = 1), immune‐mediated disease (n = 1), and gastrointestinal disease (n = 1). All CT images were assessed systematically. Muscular disruption or dislocation of the pelvic organs into the perineal area, suggestive of PH, was absent in the images.

### 3.2 Volumetric measurements

**FIGURE 3:**
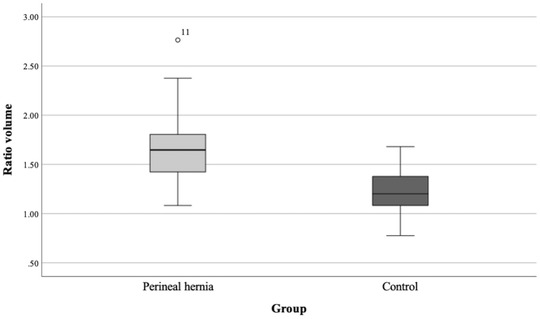
Box‐and‐whisker plots of prostatic ratio volumes in perineal hernia (n = 46) and control (n = 23) groups. For each box, the horizontal line represents the median value, and the lower and upper boundaries of boxes represent the 25th and 75th percentiles, respectively. The ratio volume in the perineal hernia group was significantly *(P < *.0001) higher than that in the control group

The mean ± SD prostatic volume was 56.0 cm^3^ ± 36.6 (range 13.0–145.6) and 31.6 cm^3^ ± 33.2 (range 1.5‐125.9) in the PH and control groups, respectively. The mean ± SD RV was 1.66 ± 0.31 (range 1.08‐2.76) and 1.23 ± 0.24 (range 0.78–1.68) in the PH and control groups, respectively. The RV was significantly (*P* < .001) larger in the PH group than in the control group (Figure 3). The estimated difference between the groups was 0.429 (95% CI 0.289–0.568). A sensitivity analysis was conducted where one obvious outlier, dog #11 from the PH group, was excluded from the model. The difference between groups remained significant (*P* < .001). Based on a 95% CI measured for RV in the control group, a lower CI of 1.13 and an upper CI of 1.34 were achieved. Prostate sizes defined by CI in both groups are presented in Table 1. In the PH group, 89.1% (41/46) of dogs had prostates that exceeded the CI of 1.34 and could thus be defined as large.

**TABLE 1:** Size of prostates in perineal hernia (PH) and control groups based on 95% confidence interval (CI) measured for ratio volume (RV) in the control group

	Control group		PH group	
Prostate size	N	Percentage (%)	N	Percentage (%)
Small (RV < 1.13)	6	26.1	1	2.2
Medium (RV 1.13‐1.34)	11	47.8	4	8.7
Large (RV > 1.34)	6	26.1	41	89.1
Total	23	100	46	100

### 3.3 Morphological and spatial assessments

**FIGURE 4:**
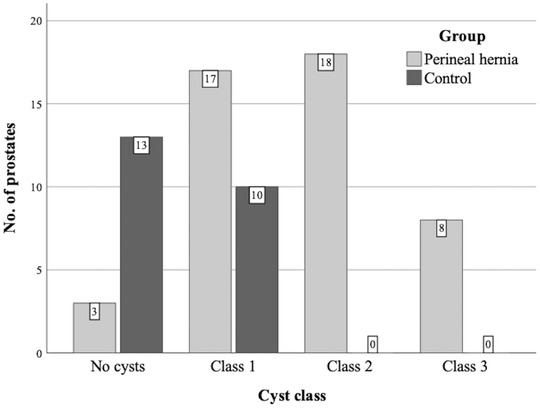
Number of intraprostatic cysts > 5 mm in diameter in prostates of perineal hernia (n = 46) and control (n = 23) groups. Prostates were placed into classes depending on the number of cysts: class 1 (1–5 cysts), class 2 (6–10 cysts), and class 3 (>10 cysts)

Heterogenicity of the prostate in CT images was evident in 97.8% (45/46) and 52.2% (12/23) of the PH and control groups, respectively. The difference between groups was significant (*P* < .001). In the PH group, the prevalence of intraprostatic cysts was 93.5% (43/46), while the control group had a significantly (*P* < .001) lower prevalence of 43.5% (10/23). Furthermore, the number of cysts in each prostate was higher in the PH group, with 17.4% (8/46) included in class 3, while all prostates in the control group were included in class 1 (Figure 4). The mean ± SD largest cyst diameter was 1.2 cm ± 0.7 (range 0.5–3.5) in the PH group and 0.7 cm ± 0.1 (range 0.6–1.0) in the control group (*P* = .013). Paraprostatic cysts were found in 17.4% (8/46) of dogs in the PH group, with a mean ± SD maximum diameter of 2.9 cm ±1.5 (range 1.6–6.4). Focal mineralizations were present in 32.6% (15/46) of dogs in the PH group, with a mean ± SD of 2.33 ± 1.4 (range 1–6) lesions per prostate. Mineralizations and paraprostatic cysts were absent in the control group.

The prostatic location was abdominal in 6.5% (3/46) and 13.0% (3/23) of dogs and pelvic in 65.2% (30/46) and 87.0% (20/23) of dogs in the PH and control groups, respectively. In the PH group, 28.3% (13/46) had herniated prostates. The mean ± SD of prostatic rotation in the PH group was 13.91**°** ± 15.41**°** (range 1.53**°**–78.23**°**) and 24.30**°** ± 20.53° (range 2.24**°**–118.77**°**) in the dorsal and transverse planes, respectively (Table 2). In the control group, the mean rotation was 4.75**°** ± 2.77**°** (range 0.7**°**–9.92**°**) and 6.85**°** ± 5.24**°** (0.85**°**–20.30**°**) in the dorsal and transverse planes, respectively. The difference between groups was significant in both dorsal (*P* = .003) and transverse (*P* < .001) planes. In both groups, prostates located in the abdomen seemed to be least rotated. In the PH group, prostates located in the hernia tended to be most severely rotated, with the largest rotation being 118.77° in the transverse plane.

**TABLE 2:** Degree of rotation of prostates in perineal hernia (PH) and control groups depending on the location of the prostate

		Degree of rotation			
		Control group (n = 23)		PH group (n = 46)	
Rotational plane	Prostate location	Mean	Range	Mean	Range
Transverse	Abdomen	5.34°	0.85–12.48°	12.19°	2.24–25.05°
	Pelvic	7.08°	2.74–20.30°	18.67°	2.26–65.31°
	Hernia	–	–	40.07°	4.87–118.77°
	Total	6.85°	0.85–20.30°	24.30°	2.24–118.77°
Dorsal	Abdomen	4.34°	3.25–5.75°	4.80°	3.51–7.27°
	Pelvic	4.81°	0.70–9.92°	10.05°	1.53–38.13°
	Hernia	–	–	24.90°	3.70–78.23°
	Total	4.75°	0.70–9.92°	13.91°	1.53–78.23°

The location of the bladder also varied between groups. In the PH group, the neck of the bladder was in the abdomen in 80.4% (37/46), in the pelvic cavity in 17.4% (8/46), and in the hernia in 2.2% (1/46) of dogs. In the control group, 95.7% (22/23) were located in the abdomen, with only 4.3% (1/23) in the pelvic cavity. A flexure of the urethra could be seen in 60.9% (28/43) of dogs in the PH group but was absent in the control group (Figure 1D).

## 4 DISCUSSION

Based on the authors’ review of the literature, this was the first published prospective study to investigate prostatic changes associated with PH using CT imaging. The findings supported both of our hypotheses. First, we found that dogs with PH had significantly larger prostates than control dogs. Second, prostates in the PH group were more often heterogeneous, contained cysts, paraprostatic cysts, and focal mineralizations, and had abnormal rotation and location. In addition, the cranio‐caudal flexure of the urethra, a common finding in the PH group, was absent in the control group.

The incidence of prostatic disease is reported to be 10–80% in dogs with PH, with the diagnosis usually based on rectal palpation, radiography, ultrasound, or a combination of these.^4^, ^6^, ^7^, ^13^, ^14^, ^15^ One study questioned the role of prostatic disease in PH formation, as only 5 of 43 dogs had a significantly enlarged prostate on radiographs.^4^ Prostates were deemed enlarged if the ratio of prostatic diameter to the pubis‐to‐sacral promontory measurement was >70. In our study, most prostates were displaced caudally, making radiographic assessment challenging due to tissue superimposition. CT imaging avoids this, providing more detail regarding both size and structure.^8^, ^9^, ^10^ In addition, we measured volume instead of one‐dimensional parameters and found the prostates in the PH group to be enlarged. A study with a similar incidence of prostatic enlargement to ours is that by Merchav et al., who prospectively investigated relaxin receptors in the perineal muscles of dogs with PH. They noted that 80% (12/15) of dogs had prostatic enlargement; however, they did not elaborate on how this diagnosis was made.^13^

There is a limited number of studies assessing canine prostatic volume on CT images, this being the first to do so in dogs with PH. Benign prostatic hyperplasia (BPH) is common in intact male dogs and is thought to be the underlying cause of most prostatic diseases.^8^ Prostatic volume varies with age and breed, and to date, there are no published reference values in dogs.^8^, ^16^ Haverkamp et al. measured prostatic volume using the Amira® program and correlated it with the size of the dog using the length of L6. The authors reported the mean RV of intact male dogs (n = 58) to be 1.3.^12^ This is in accordance with the RV in our control group (1.23). Haverkamp et al. also found that the group with the largest prostates, the cystic group, had an RV of 1.4.^12^ In our study, the average RV was even higher in the PH group (1.66). When the CI of prostatic RV in our control group was used to set reference values for small, normal, and large prostates, we found that 89.1% of dogs in the PH group had a large prostate. Further studies of RV with a larger sample size of age‐matched intact dogs are needed to verify normal reference ranges.

The presence of intraprostatic or paraprostatic cysts and mineralization in the prostate of the dog implies a more severe pathologic process than simple enlargement.^8^, ^9^, ^17^ Prostatic cysts are the accumulation of prostatic fluid caused by obstruction of canaliculi, often associated with BPH. These cysts are initially microscopic, but as the pathology escalates, the cysts connect, forming macroscopic cysts of various shapes and sizes.^17^ Paraprostatic cysts have been described as large, thin‐walled cysts attached to the prostate or have been associated with remnants of embryonic tissue or protruding prostatic retention cysts. They are considered to be rare, although the prevalence is unknown.^17^, ^18^ Prostate mineralizations are typically small, focal lesions that can be associated with prostatic disease, especially prostatic neoplasia.^19^, ^20^

Haverkamp et al. found that 84.8% of dogs aged over 4 years had prostatic cysts in CT images,^12^ which is a larger proportion than in our control group (43.5%) and closer to that of our PH group (93.5%). This finding can partly be explained by the difference in the diameter of the lesion defined as a cyst, which in our study was > 5 mm and in the Haverkamp study was only > 1.2 mm.^12^ Mantziaras et al. examined the prostates of 1003 male dogs aged 1–18 years with ultrasound and found prostatic cysts in 33.6%, paraprostatic cysts in 1.6%, and mineralization in 7% of intact male dogs.^21^ These findings, considering the age distribution, are similar to our control group.

In dogs with PH, prostatic morphology has been assessed even less than size,^7^, ^15^ as palpation, visualization, and radiography are unreliable methods for intraprostatic assessment.^9^ A study by Brissot el al. detected prostatic disease via ultrasonography or visualization in 51.2% of dogs with PH, with histologic confirmation in 41.5%. Only seven dogs had intra‐ or paraprostatic cysts, and five had abscesses.^7^ In our study, almost all dogs had intraprostatic cysts (93.5%), while paraprostatic cysts were diagnosed in 17.4%. This large difference between our findings and those of previous studies can partially be explained by the retrospective nature of these studies and the differing methods of diagnosis, which varied from ultrasound to visualization.

In the PH group, focal prostatic mineralizations were common (32.6%). The disease process underlying prostatic mineralization in intact male dogs is unclear, although mineral accumulations in the walls of prostatic cysts are suspected. An association has been drawn between neoplasia and mineralization in the prostates of castrated dogs; however, in intact male dogs, prostatic cancer is uncommon.^19^, ^20^

In dogs with PH, herniation of the prostate is relatively common, occurring in 4–40% of PH cases,^4^, ^6^, ^7^ which is consistent with our finding of 28.3%. In dogs, the location of the prostate typically changes with age, migrating from an intrapelvic to abdominal site as the prostate grows.^8^, ^20^ Although the dogs of our PH group had enlarged prostates, only 6.5% of prostates were in the abdominal cavity, while 65.2% were in the pelvic cavity. We suggest that this is due to increased pressure from the abdominal cavity as the dog struggles to defecate, pushing the prostate caudally into the pelvic cavity and hernia.

Based on the authors’ review of previous literature, rotation of the prostate in dogs with PH has not been previously reported. While assessing CT images of the prostates in the PH group, it became visually obvious that prostates were often severely rotated in relation to the midline of the dog. In the transverse plane, the mean rotation was 24.30**°** in the PH group and 6.85° in the control group; thus, a significant difference was present. Based on our results, we suggest that the prostates of dogs with PH have increased mobility as part of the disease process.

In dogs with PH, the incidence of bladder retroflexion has been reported to be approximately 18–29%.^7^, ^14^, ^15^, ^22^ Maute et al. surgically treated PH with laparotomy in 32 dogs and noted that up to 22% of dogs had bladder retroflexion. Additionally, 31% of dogs had changes in the serosal surface of the bladder indicative of previous displacement.^14^ In our study, only one dog had bladder retroflexion in CT images; however, in 17.4%, the neck of the bladder was located in the pelvic cavity. In our PH group, 28.3% of prostates were herniated and could have effectively pulled the bladder caudally. Previous retroflexion of the bladder prior to CT imaging cannot be ruled out. Dogs with bladder retroflexion are considered emergency cases and are usually surgically treated as soon as possible, while dogs referred to this study typically had to wait at least a few days to take part in the study. This can partially explain the low occurrence of bladder retroflexion in our study.

An S‐shaped flexure of the urethra was found in 60.9% of PH dogs, an anomaly that, to the authors’ knowledge, has not been described in previous literature on PH. Although the incidence of urethral flexures in male dogs is thought to be uncommon, in bitches, it is associated with the intrapelvic location of the bladder.^23^, ^24^ In healthy dogs, the pelvic location of the bladder neck can be an incidental finding, although it has been associated with urinary incontinence.^25^ Urinary obstruction caused by a flexure of the urethra has been reported in three male Yorkshire terriers with pelvic bladders.^23^, ^24^ We suggest that the relatively high incidence of urethral flexures in dogs with PH is due to the caudal displacement of the prostate toward or into the hernia.

The hormone relaxin has been suggested to play a role in the etiopathogenesis of PH.^5^, ^13^ In male dogs, relaxin is mainly of prostatic origin, with relaxin immunoreactivity markedly increased in prostatic and paraprostatic tissue of dogs with PH.^5^ In addition, significantly higher expression of relaxin receptors occurs in the perineal muscles of dogs with PH than in healthy intact dogs.^13^ It has been proposed that relaxin leaks from a cystic prostate into the surrounding tissues, leading to perineal muscle atrophy.^5^ Our findings of large, cystic prostates in dogs with PH support this theory, indicating that prostatic changes are associated with PH. It is unclear why only some dogs with cystic BPH develop PH. PH is a naturally occurring disease that cannot be experimentally induced. Determining the exact etiopathogenesis of PH is thus challenging.

This study had some limitations. Assessment of prostates was based solely on CT imaging, and the study design did not include cytological or histological analysis of the prostates. As such, cytological and histological differentiation between cysts and abscesses and diagnosis of pathologies, such as BPH, prostatitis, and tumors, could not be performed. The purpose of this study, however, was to compare the prostatic changes of PH dogs and age‐matched male dogs on CT images, not to differentiate between disease processes. Several dogs within the control group had enlarged and cystic prostates; however, these changes were significantly more severe in the PH group.

Another limitation was the retrospective nature of the control group collected from the VTHUH database. Although technical CT parameters were standardized in the PH group, the parameters in the control group varied depending on the protocol used, and not all dogs received contrast medium. Based on unpublished data used with permission from Hanna Salonen, University of Helsinki, measuring the prostate in noncontrast images does not affect the reliability of the measurement. In addition, different technical parameters, such as protocol and slice thickness under 2.5 mm, do not affect the reliability of prostate volume measurements in normal dogs. The volume measurements in severely changed prostates may be more challenging since prostatic tissue may be difficult to discern from surrounding tissue, such as the rectum and adjacent muscles. To avoid the risk of volume overestimation, we were careful to exclude all tissue and cysts not obviously connected to the prostates. Heterogenicity and small cysts are easier to detect when using contrast medium; the lack of a contrast agent in the control group may have affected our results. We chose to include cysts that were >5 mm in size to minimize this risk, as smaller cysts can be more easily overlooked. Paraprostatic cysts, mineralizations, and rotation and location of the prostate should not be significantly affected by use of a contrast agent. Additionally, the formerly mentioned unpublished data show that the repeatability of volumetric measurements is high, which is why volumetric measurements were performed only once.

In conclusion, these findings support the use of CT as an adjunct imaging modality for the evaluation of prostatic changes in PH dogs. In dogs with PH, prostatic changes on CT images significantly differed from changes in control dogs. These findings can be used to justify recommending castration in conjunction with herniorrhaphy for dogs with PH. Further studies are needed to evaluate nonprostatic CT findings in the pelvic cavity of PH dogs.

## LIST OF AUTHOR CONTRIBUTIONS

### Category 1

(a) Conception and Design: Åhlberg, Salonen, Mölsä, Laitinen‐Vapaavuori
(b) Acquisition of Data: Åhlberg, Salonen, Mölsä
(c) Analysis and Interpretation of Data: Åhlberg, Mölsä

### Category 2

(a) Drafting Article: Åhlberg
(b) Revising Article for Intellectual Content: Åhlberg, Salonen, Mölsä, Laitinen‐Vapaavuori

### Category 3

(a) Final Approval of the Completed Article: Åhlberg, Salonen, Mölsä, Laitinen‐Vapaavuori

### Category 4

(a) Agreement to be accountable for all aspects of the work in ensuring that questions related to the accuracy or integrity of any part of the work are appropriately investigated and resolved: Åhlberg, Salonen, Mölsä, Laitinen‐Vapaavuori

## CONFLICT OF INTEREST

The authors have declared no conflict of interest.

## Supporting information

Supporting Information
