# Supplementary material for: CT imaging of dogs with perineal hernia reveals large prostates with morphological and spatial abnormalities
Source: Vet Radiol Ultrasound. 2022 Mar 29;63(5):530–8. doi: 10.1111/vru.13087 (PMC9790208; doi:10.1111/vru.13087)
Supplement: Supplementary file 1 — Supporting Information [file VRU-63-530-s001.docx]

**Supplement 1.** Technical parameters of computed tomography scans for dogs in the control group.

| Dog # | Contrast agent and phase | Slice thickness (mm) | Voltage  (kV) | Current  (mAs) | Pitch | Speed (mm/rot) | Rotation time (s) | Noise index |
| --- | --- | --- | --- | --- | --- | --- | --- | --- |
| 1 | Yes: interstitial | 1.25 | 140 | 320-601 | 0.984:1 | 39.37 | 0.5 | high |
| 2 | Yes: interstitial | 0.625 | 120 | 120 | 0.516:1 | 20.62 | 0.8 | low |
| 3 | Yes: portal | 0.625 | 120 | 100 | 0.984:1 | 39.37 | 0.5 | low |
| 4 | Yes: portal | 0.625 | 120 | 100 | 0.984:1 | 39.37 | 0.5 | low |
| 5 | Yes: portal | 0.625 | 120 | 367-387 | 0.984:1 | 39.37 | 0.5 | low |
| 6 | Yes: interstitial | 0.625 | 120 | 247-378 | 0.984:1 | 39.37 | 0.4 | high |
| 7 | Yes: interstitial | 0.625 | 120 | 292-476 | 0.984:1 | 39.37 | 0.5 | high |
| 8 | Yes: interstitial | 2.5 | 120 | 231-326 | 0.516:1 | 20.62 | 0.5 | high |
| 9 | Yes: interstitial | 0.625 | 120 | 87-111 | 0.516:1 | 20.62 | 0.6 | high |
| 10 | Yes: interstitial | 0.625 | 100 | 99-104 | 0.516:1 | 20.62 | 0.8 | low |
| 11 | Yes: portal | 0.625 | 120 | 257-275 | 0.984:1 | 39.37 | 0.5 | low |
| 12 | Yes: interstitial | 0.625 | 100 | 100-102 | 0.516:1 | 20.62 | 0.8 | low |
| 13 | Yes: interstitial | 0.625 | 120 | 80 | 0.984:1 | 39.37 | 0.8 | low |
| 14 | Yes: portal | 0.625 | 120 | 302-311 | 0.984:1 | 39.37 | 0.5 | low |
| 15 | No | 0.625 | 120 | 229-383 | 0.516:1 | 20.62 | 0.8 | low |
| 16 | No | 0.625 | 120 | 80 | 0.516:1 | 20.62 | 0.6 | high |
| 17 | No | 0.625 | 120 | 89-92 | 0.516:1 | 20.62 | 0.8 | low |
| 18 | No | 0.625 | 120 | 139-140 | 0.516:1 | 20.62 | 0.6 | high |
| 19 | No | 0.625 | 120 | 113-126 | 0.516:1 | 20.62 | 0.6 | high |
| 20 | No | 0.625 | 120 | 202-204 | 0.516:1 | 20.62 | 0.6 | high |
| 21 | No | 0.625 | 120 | 139-148 | 0.516:1 | 20.62 | 0.6 | high |
| 22 | No | 0.625 | 120 | 80 | 0.516:1 | 20.62 | 0.6 | high |
| 23 | No | 0.625 | 120 | 669 | 1.375:1 | 55.00 | 0.4 | high |
